# Ultrawideband Polarization-Independent Nanoarchitectonics: A Perfect Metamaterial Absorber for Visible and Infrared Optical Window Applications

**DOI:** 10.3390/nano12162849

**Published:** 2022-08-18

**Authors:** Mohammad Lutful Hakim, Abu Hanif, Touhidul Alam, Mohammad Tariqul Islam, Haslina Arshad, Mohamed S. Soliman, Saleh Mohammad Albadran, Md. Shabiul Islam

**Affiliations:** 1 Pusat Sains Ankasa (ANGKASA), Institut Perubahan Iklim, Universiti Kebangsaan Malaysia (UKM), Bangi 43600, Selangor, Malaysia; 2 Department of CSE, International Islamic University Chittagong (IIUC), Kumira, Chattogram 4318, Bangladesh; 3 Department of Electrical, Electronic and Systems Engineering, Faculty of Engineering and Built Environment, Universiti Kebangsaan Malaysia (UKM), Bangi 43600, Selangor, Malaysia; 4 Electrical Engineering Department, College of Engineering, University of Ha’il, Ha’il 81481, Saudi Arabia; 5 Center for Artifcial Intelligence and Technology, Facult of Information Science and Technology, Universiti Kebangsaan Malaysia (UKM), Bangi 43600, Selangor, Malaysia; 6 Department of Electrical Engineering, College of Engineering, Taif University, P.O. Box 11099, Taif 21944, Saudi Arabia; 7 Department of Electrical Engineering, Faculty of Energy Engineering, Aswan University, Aswan 81528, Egypt; 8 Faculty of Engineering (FOE), Multimedia University, Persiaran Multimedia, Cyberjaya 63100, Selangor, Malaysia

**Keywords:** metamaterial absorber, polarization-independent, optical window, nanoarchitectonics

## Abstract

This article presents numerical analysis of an ultrathin concentric hexagonal ring resonator (CHRR) metamaterial absorber (MMA) for ultrawideband visible and infrared optical window applications. The proposed MMA exhibits an absorption of above 90% from 380 to 2500 nm and an average absorbance of 96.64% at entire operational bandwidth with a compact unit cell size of 66 × 66 nm^2^. The designed MMA shows maximum absorption of 99% at 618 nm. The absorption bandwidth of the MMA covers the entire visible and infrared optical windows. The nickel material has been used to design the top and bottom layer of MMA, where aluminium nitride (AlN) has been used as the substrate. The designed hexagonal MMA shows polarization-independent properties due to the symmetry of the design and a stable absorption label is also achieved for oblique incident angles up to 70 °C. The absorption property of hexagonal ring resonator MMA has been analyzed by design evaluation, parametric and various material investigations. The metamaterial property, surface current allocation, magnetic field and electric field have also been analyzed to explore the absorption properties. The proposed MMA has promising prospects in numerous applications like infrared detection, solar cells, gas detection sensors, imaging, etc.

## 1. Introduction

Metamaterials are artificially structured composite materials with unusual properties that cannot be obtained naturally [1,2]. The geometry and size of the structure can be adjust to achieve desired permeability property of the metamaterial [3], which makes metamaterial suitable for sensing [4,5,6,7,8], absorption [9,10,11], reflect array [12,13,14], the antenna [15,16,17] and detection [18]. Metamaterials are also employed to monitor the low intensities of different gases in various applications, from environmental monitoring to home safety monitoring systems [19,20]. Within this perspective, metamaterials have a good recognition in optics and photonic devices [21,22,23]. Researchers are currently working on MMA design for visible to the infrared regime, which is utilized in various applications [24]. Most of the MMAs are designed the visible regimes and constrained by the limited absorption spectrum, which restricts their uses to thermal emission manipulations and nanoantenna resonators [25,26,27]. Wideband absorbers are very attractive for applications like solar energy converters, thermal emitters, and artificial colors with angle and polarization-insensitive features [28,29,30]. The near-unity absorption is achieved by electromagnetic (EM) wave-inspired modulation of the interaction between matter and light [23].

EM wave absorbers are categorized into two types, i.e., resonant absorbers [11] and wideband absorbers [23,31]. According to anomalous diffraction theory, the resonant absorbers are based on the resonance interaction between light and materials at their working frequencies [3]. Wideband absorbers depend on those materials whose characteristics are unrelated to the frequency with the highest intrinsic loss [25]. Multiple methods have been reported to achieve a wide band property, such as perpendicularly standing nanowires, multiple resonators employing in-plane arrangement, multilayer vertically stacked resonant elements etc. [32]. These methods broaden the absorption bandwidth; however, suffer from structural and manufacturing complexities. The merging of multilayer absorbers is challenging with industrial technologies due to its larger size, high cost, and use of new material [29]. Notably, the building process of multiple resonators is cost-efficient compared to the multilayer method. Therefore, designing an absorber with a wideband absorption spectrum, simple fabrication and low cost is necessary for optical window applications. The use of different materials is another potential method opted by numerous researchers to develop broadband absorbers. The idea of using Nickel (Ni) is one of the essential parts of this sequence due to its outstanding optical and chemical characteristics to develop a wideband absorber. The top layer’s geometrical shape can provide broad absorption and wide-angle stability. In [20], a Ni-base wideband metamaterial absorber was designed and investigated its performance in visible and near-infrared regimes. Aluminium Nitride was selected as the substrate material because of its suitable semiconductor property with stable wurtzite (WZ) and attractive high thermal conductivity, which prevent attenuation during high temperatures.

In this paper, a simple, efficient, and wideband metamaterial absorber has been designed for the visible and infrared region by using Ni and Aluminium Nitride. The proposed structure comprises a concentric hexagonal ring resonator. Near-perfect absorption shows by the design for the incident TM (Transverse Magnetic) and TE (Transverses Electric) waves. Due to the symmetrical patch design, polarization-independent oblique incident-angle stability shows up to 70 °C. These attributes make the designed absorber a potential candidate for sensing, detection applications, and energy harvesting, and can be employed to monitor the low intensities of different gases in various applications, from environmental monitoring to home safety monitoring systems.

## 2. MMA Design

**Figure 1:**
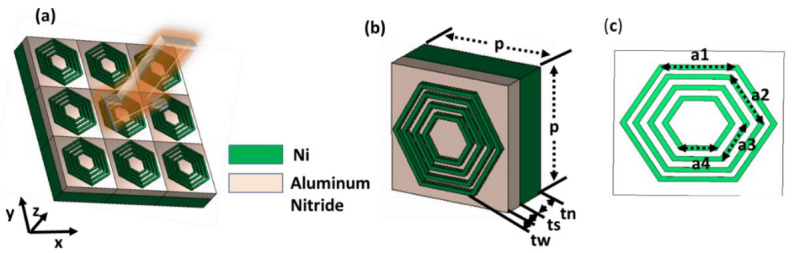
MMA unit cell design (**a**) 3 × 3 array, (**b**) perspective view, (**c**) front view.

The proposed metamaterial absorber comprises three layers configuration (metal-dielectric–metal), shown in Figure 1. The perspective views of 3 × 3 metamaterial absorbers are displayed in Figure 1a. The design parameters are illustrated in Figure 1a,b. The top and bottom layers are Nickel (Ni) metal, and Aluminium Nitride (AlN) is used as the dielectric substrate with a dielectric constant of 8.6 and an electrical loss tangent of 0.0003.

**Figure 2:**
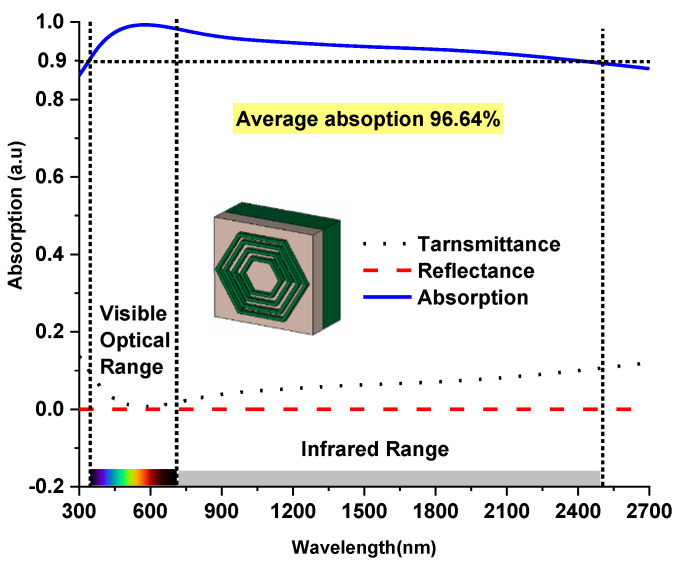
The proposed MMA’s absorption, reflectance and transmittance curves.

The AlN material is chosen over other substrate materials due to its attractive high thermal conductivity (200 W/K/m) and since it does not attenuate during high temperatures. Moreover, AlN is a semiconductor with a stable wurtzite (WZ) structure, which is a very significant property for optoelectronic applications in a large spectral region. The unit cell upper layer consists of six concentric hexagonal-shaped ultrathin resonators. As indicated in Figure 1, P = 66 nm is the of the unit cell optimized boundary length. The four equivalent hexagonal rings have side-arm lengths of a1 = 30 nm, a2 = 25 nm, and a3 = 20 nm, a4 = 15 nm, respectively. The widths of the hexagonal shape ring are Wd = 3 nm. In addition, tn, ts and tw represent the bottom ground, middle dielectric substrate and top patch thicknesses, respectively, where the values of tn, ts and tw are 20 nm, 12 nm and 3 nm, individually. The CST Microwave Studio has been used to design and simulated the proposed metamaterial absorber [33]. The unit cell boundary conditions have been applied along the x and y-axis. The negative z-axis is the direction of wave propagation, where open add-space boundary conditions have been defined. The total absorption (A) of the resonators was calculated by using the simplified Equation (1) [34]:

(1) A(λ)=1−T(λ)−R(λ)

where A(λ), T(λ) and R(λ) are total absorbances, transmittance and reflectance, respectively. Figure 2 illustrates the simulation result of the MMA, which demonstrates an above 92% absorption rate from a wavelength range of 380–2500 nm, which covers the entire visible and infrared optical window. The equation can also be correlated with the S parameters as $A=1-{\left|S_{11}\right|}^{2}-{\left|S_{21}\right|}^{2}$ [6], where S_11_ and S_21_ are the reflection and transmission coefficients of the absorber, respectively. Figure 2 presents the proposed design’s transmittance, reflectance and absorption curve, where above 90% absorption appeared from 380–2500 nm wavelength with an average absorption of 96.64%.

## 3. MMA Design Analysis

**Figure 3:**
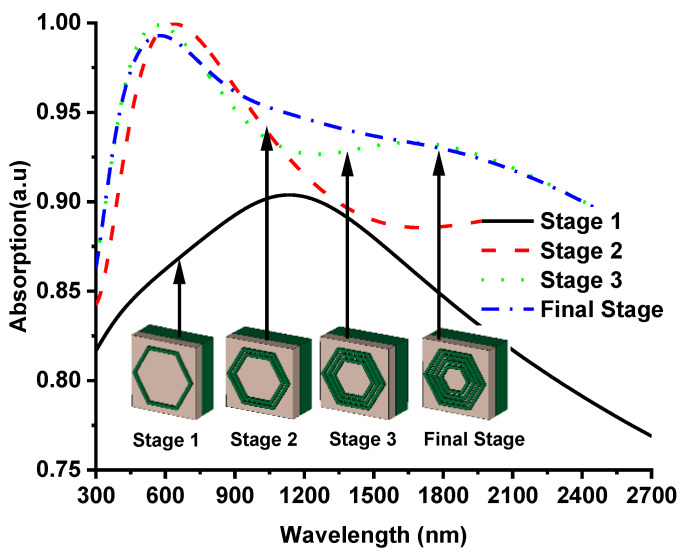
Absorption for four different stages at normal incidence.

The design evaluations of the proposed absorber have been discussed to understand the absorption performance of the different design stages. Figure 3 illustrates the absorption characteristics of the four different steps of the designed hexagonal ring resonator. Firstly, one single hexagonal ring was used and achieved low absorption at visible and far-infrared wavelengths. After that, another hexagonal ring was used inside the first ring to achieve a better absorption, which shows 99% absorptivity at visible optical wavelength. This excellent high absorption has been achieved due to the coupling capacitance between this hexagonal ring and the addition inductivity by the second ring. Later, three hexagonal rings were used to increase the absorption, which resulted in the additional coupling capacitance and inductance to increase the absorption. Therefore, the smaller rings are responsible for the absorption at the lower wavelength region, and larger hexagonal rings gradually provide higher absorption at the higher wavelength region. The design concept reflects the proportional relationship between the operational wavelength and resonator size.

**Figure 4:**
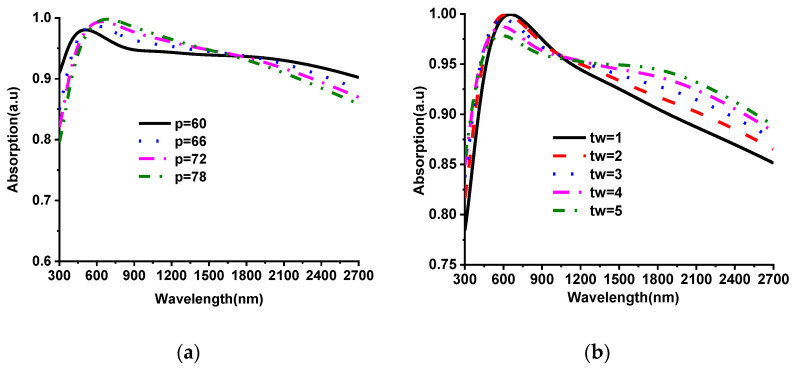

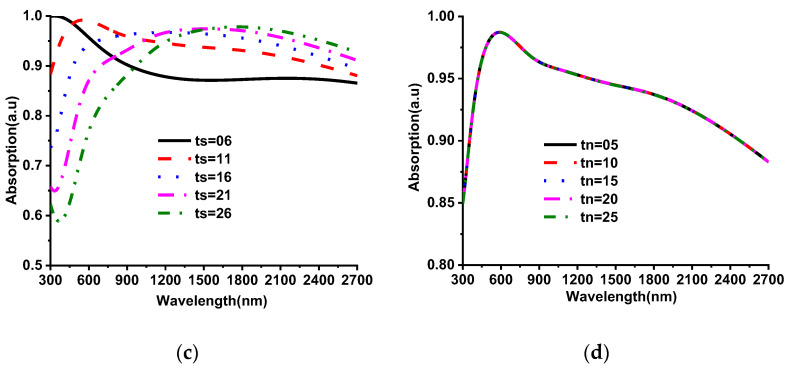
The absorption performance of different design parameters. (**a**) unit cell period (p); (**b**) height of the top of the hexagonal ring resonator (tw); (**c**) height of the substrate (ts); (**d**) the height of the bottom layer (tn).

The analysis of the MMA design parameters has been presented to understand the absorption sensitivity of parametric variation. Figure 4 presents the absorption analysis for different design parameters. The total dimension of the unit cell or periodicity of the structure is investigated by changing “P” from 60 to 78 nm with a 6 nm interval shown in Figure 4a. The value “P” suppressing shows better absorption for visible optical-region wavelength than the infrared-region wavelength. For the increment of periodicity, the absorption value increases in the near-infrared region and decreases in the far-infrared region. This occurs due to the coupling of two adjacent unit cell structures. Moreover, the absorption level and absorption bandwidth of the MMA is significantly influenced by its thickness. The top, middle- and bottom-layer thickness investigations are presented in Figure 4b–d, individually. Figure 4b illustrates the absorption for different resonator heights at the top surface, whereas the height increases by 1 nm from 1 to 5 nm. It shows good average absorption, but the visible region did not achieve a good absorption level compared to the infrared region for the increment of “tw”. This occurs due to the increment of thickness “tw”, which increases the overall size of resonators. The high absorption shifts towards a higher wavelength with the increment of resonator size. The lower value of the “tw” shows almost 100% absorption for the visible region. However, the absorption level reduces by less than 90% for the infrared regions due to the proportional relationship of resonator size and operating wavelength Figure 4c investigates the absorption by varying the substrate thickness “ts”. With the increment of ts from 11 nm to 16 nm, the overall absorption value decreases; however, the absorption percentage increases in the infrared region. On the other hand, with the suppression of the thickness (11 to 6 nm), absorption is high for the visible range. This phenomenon is also related to the size and wavelength relation; if the size of the unit cell decreases, high absorption shifts towards a lower wavelength. On the other hand, high absorption shifts towards a higher wavelength with an increment of unit cell size. The EM wave transmission through the MMA unit cell is blocked by Nickel material. The EM wave blocking capacity can be assumed by skin depth $\delta =\sqrt{\rho /\pi f\mu }=\sqrt{\rho \lambda /\pi \mu }$, where the wavelength is proportional to skin depth. Hence, to block the larger wavelength a higher skin depth is necessary [35]. The value of bottom-layer thickness “tn” that was investigated from 5 nm to 25 nm is shown in Figure 4d, where it did not affect the absorption curve because the lowest values of nickel thickness are above the skin depth.

**Figure 5:**
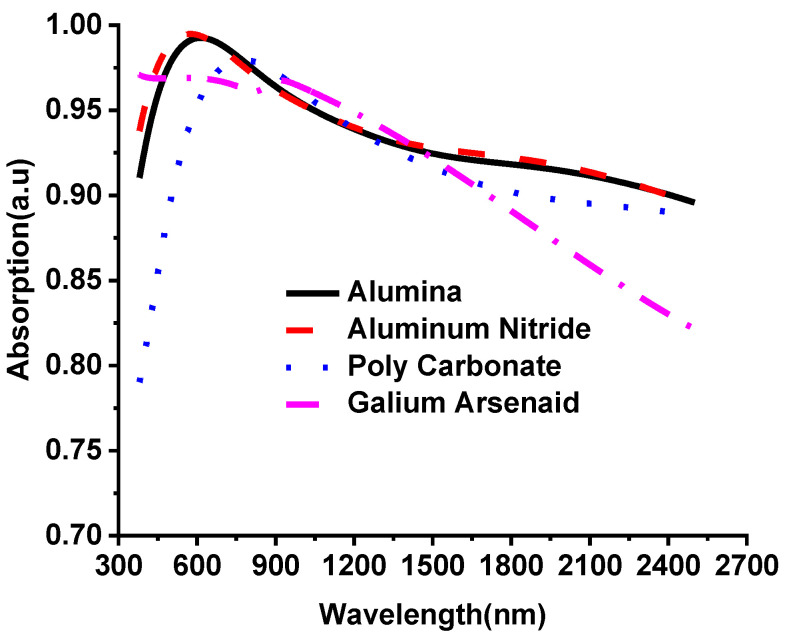
The performance investigation of the designed metamaterial absorber for different substrate materials.

Figure 5 shows that the proposed metamaterial absorber performance has been investigated with different materials. The absorption spectra are analyzed at the different dielectric, as illustrated in Figure 5. Alumina, gallium arsenide, polycarbonate and aluminium nitride were chosen to examine the absorption rate of the proposed design. It is seen that the absorption spectrum for aluminium nitride (AlN) shows better absorption at the desired wavelength range from the visible to infrared region. Alumina has similar absorption to AlN but decreases in the visible optical region. However, polycarbonate shows less absorption in the visible optical region, which is not computable with the proposed work. However, AlN shows better absorption for both visible and near-infrared spectrum for this design due to its material characteristics such as dielectric constant, electrical tangent, thermal conductivity and unit cell geometrical pattern.

## 4. Result Analysis

**Figure 6:**
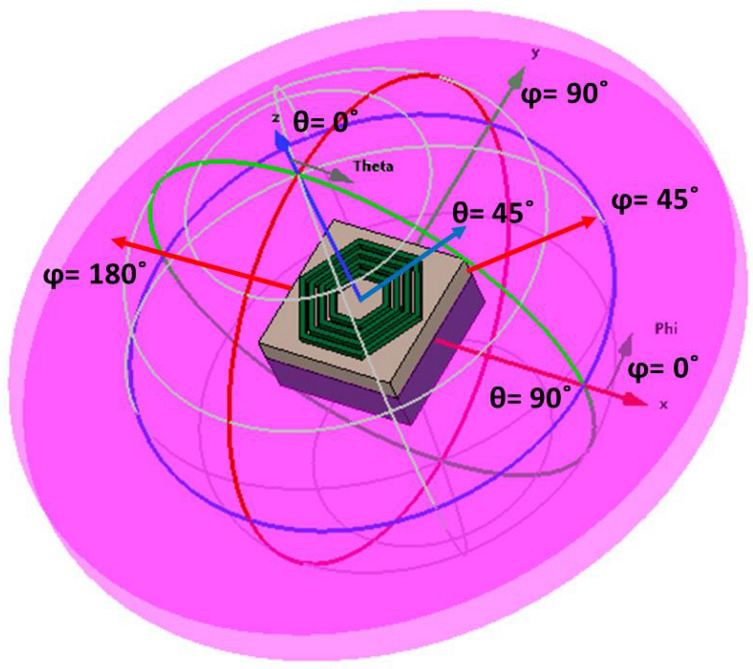
Indication of phi and theta angle of incident EM wave on MMA.

**Figure 7:**
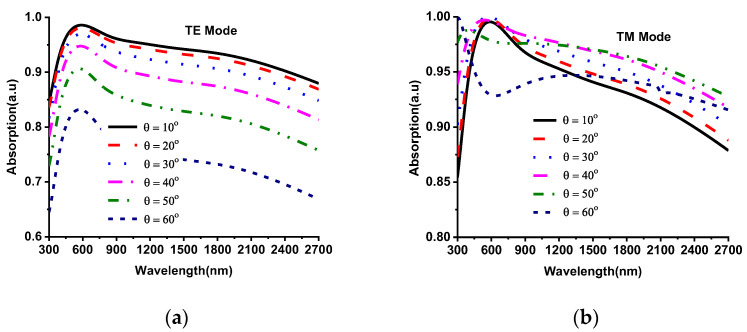
Absorption of different oblique incident angles (**a**) TE and (**b**) TM polarization.

**Figure 8:**
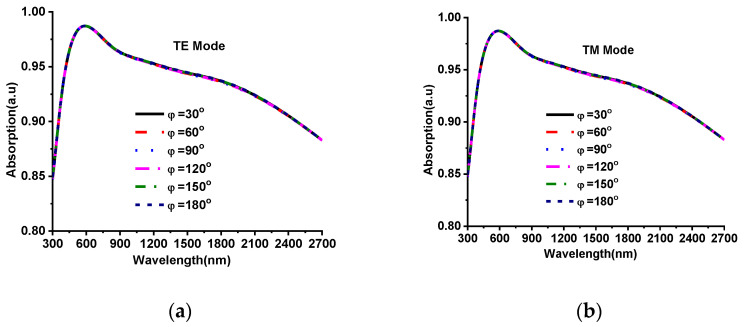
Absorption of different polarization incident angles (**a**) TE and (**b**) TM polarization.

The robustness of the designed absorber was analyzed for different oblique incident angles of EM waves (both TM and TE polarization). The incident and refraction angles have a substantial impact on the reflection coefficient. The polarization angle (phi) and oblique incident angle (theta) of the incident EM wave on MMA is illustrated in Figure 6, where EM wave propagation is towards the negative z-axis. The green half-circle indicates the 180° oblique incident-angle area of EM wave theta (θ), and the blue circle shows the 360° phi (ϕ) angle area of EM wave components such as electric and magnetic field vectors. The oblique incident angle’s stability and absorption level of the designed MMA were analyzed by changing the oblique angle incidences from 0° to 90°. The absorption property for the oblique incident angle of TM and TE polarization are revealed in Figure 7. A high absorption at normal incident θ = 0°, and a minor variation is observed in absorption level at low oblique incidents up to θ = 30° for both polarization. However, for the larger oblique incidents (θ ≥ 40°), the absorption of TE polarization starts fluctuating, as shown in Figure 7a. On the other hand, the absorption plot of TM polarization for oblique incidents is shown in Figure 7b. Apart from fluctuations, high absorption is still maintained by designed MMA up to 90% for the total bandwidth at oblique incidences from 10°–40°. It goes down from 90% at θ ≥ 50°; however, designed MMA shows over 70% absorption for the TE wave polarization and maintains over 85% absorption for TM wave polarization. This indicates that the proposed Ni-AlN-Ni-based MMA is suitable for various applications due to its stable absorption of 70% at different oblique incident angles. The polarization-angle insensitivity is also an expected performance index in the applied application fields. The proposed MMA shows polarization-angle (ϕ) insensitivity due to its rotational symmetrical patch structure. Figure 8a,b show the absorption curve of various polarization angles for TE and TM polarization. The polarization angles varied from 0°–180° by increasing 30°. The MMA shows a uniform absorption, and no fluctuation is observed for various polarization angles (ϕ) for both TE and TM polarization. Therefore, the proposed Ni-AlN-Ni-based MMA achieved a broad absorption band by fulfilling the polarization-independent condition.

**Figure 9:**
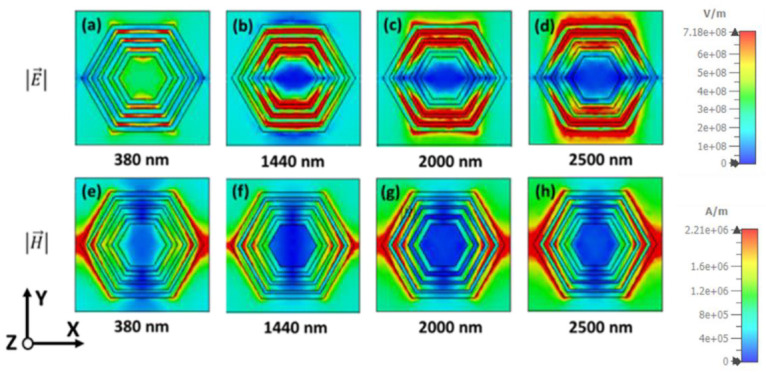
The electric field |E|, (**a**) 380 nm, (**b**) 1440 nm, (**c**) 2000 nm and (**d**) 2500 nm. The magnetic field |H| distribution of TE polarization (**e**) 380 nm, (**f**) 1440 nm, (**g**) 2000 nm and (**h**) 2500 nm.

**Figure 10:**
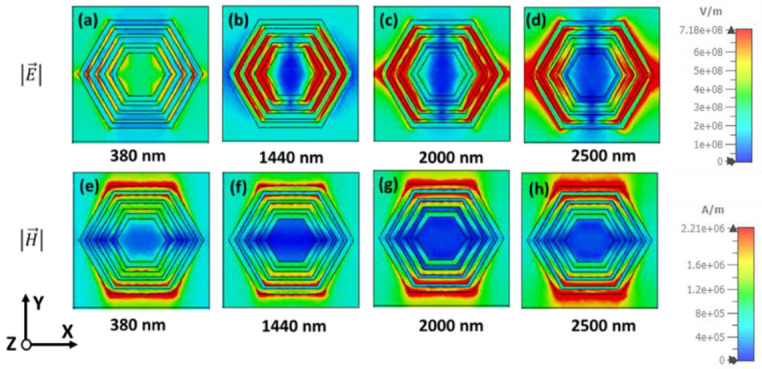
The electric field |E|, (**a**) 380 nm, (**b**) 1440 nm, (**c**) 2000 nm and (**d**) 2500 nm. The magnetic field |H| distribution of TM polarization (**e**) 380 nm, (**f**) 1440 nm, (**g**) 2000 nm and (**h**) 2500 nm.

The magnetic field $\left|\vec{H}\right|$ and electric field $\left|\vec{E}\right|$ density of TE polarization for different wavelengths are presented in Figure 9 to understand the absorption behavior of the projected MMA. Figure 9a–d illustrates the electric field at wavelengths 380 nm, 1440 nm, 2000 nm, and 2500 nm, respectively. As the operating wavelength increases, the electric-field density increases like a vertical dipole-shape pattern. It shows the high electric-field intensity on both sides of the hexagonal ring-shaped resonator. A potential electric-field density is observed towards the outer ring at higher wavelengths, which leads to high absorption due to the surface plasmon polariton. This phenomenon happens because the outer ring is responsible for higher absorption at the upper wavelength region. The surface plasmon happens due to the conduction of electrons between dielectric and metal at the surface, where it conducts strongly with electromagnetic radiation. Also, the electric field intensity is particularly confined on the outer hexagonal resonator and observed a dipole-type pattern. Thus, the smooth electric-field distribution between the hexagonal ring-shaped is formed by the mutual coupling of multiple Plasmon’s. Moreover, AlN material has a large dielectric constant compared to other metals and plays a vital role in absorptivity. Therefore, it also influences the high absorption in the near and mid-infrared bands. Correspondingly, in Figure 9e–h, the magnetic-field intensity also gradually increases along the wavelength, high intensity is seen in the horizontal of the MMA, and the magnetic-field intensity gradually moves to the outer ring from the inner ring. The electric- and magnetic-field distribution of TM polarization is also presented in Figure 10, which shows similar behavior at TE polarization, but in 90-degree rotation. Therefore, the high wideband absorption of the proposed Ni-AlN-Ni ultrathin concentered hexagonal ring structure MMA originates from the combination of coupled results of multiple plasmons, SPP and the material itself.

**Figure 11:**
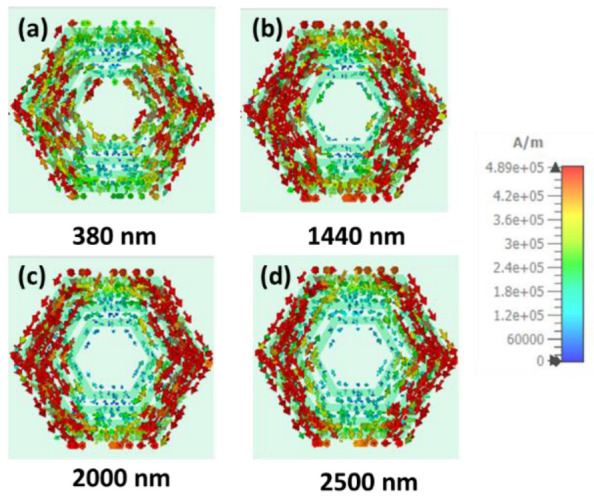
Surface current distribution of TE polarization for four operating wavelengths (**a**) 380 nm, (**b**) 1440 nm, (**c**) 1980 nm, (**d**) 2500 nm, gradually.

**Figure 12:**
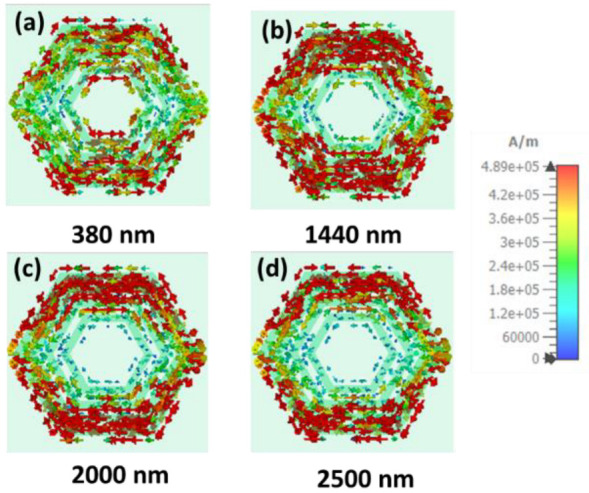
Surface current distribution of TM polarization for four operating wavelengths (**a**) 380 nm, (**b**) 1440 nm, (**c**) 1980 nm, (**d**) 2500 nm, gradually.

Moreover, the surface current has been analyzed at different operating wavelengths. Figure 11 and Figure 12 show the top layer surface current density of TE and TM polarization, respectively, at 380 nm, 1440 nm, 2000 nm, and 2500 nm. The surface current has been mainly confined on the top concentrated hexagonal ring-shaped resonator surface, leading to the electric resonance. Therefore, the absorption appears because of the electric resonance.

The impedance matching method is one of the crucial parameters for understanding absorption behavior, where the impedance matching among the optimal absorber and the surrounding medium is vital. The proposed absorber’s effective impedance (Z_λ_) is calculated by Equation (2) [36], which shows the relation between S_11_, S_21_, and Z_λ_. The real and imaginary part of Z_λ_ is denoted by Re(Z) and Im(Z) in Equation (3), respectively. Moreover, the spectral absorptance (α_λ_) of the absorber is expressed by Equation (4).

(2) $$Z_{\lambda }=\sqrt{\frac{{\left({1+S}_{11}\right)}^{2}-S_{21}^{2}}{{\left(1-S_{11}\right)}^{2}-S_{21}^{2}}}$$

(3) Zλ=Re(Z)+Im(Z).i

(4) αλ=4Re(Z)[1+Re(Z)]2+[Im(Z)]2

**Figure 13:**
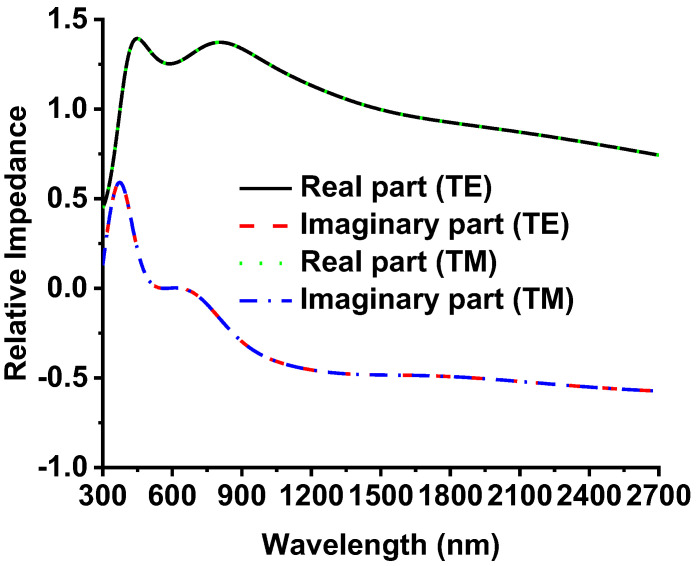
The TM- and TE-wave normalized impedance of the proposed MMA absorber.

According to Equation (4), when the absorber’s normalized impedance matches with the free-space impedance (Z = Z_o_ =1), the perfect absorption has been attained. In other words, the closer the imaginary part is to zero, and the closer the real part is to one, the higher the absorption of the MMA. The relative impedance for the proposed MMAs’ TM and TE wave polarization is presented in Figure 13. There is no significant or small change between TM and TE polarization impedance due to the symmetricity of the MMA patch. The wavelength 380 nm to 2700 nm shows that the impedance of the optical absorber matches the surrounding impedance (free space). The designed absorber realized high absorption of around 99% in the visible region. The rest of the wavelength varies slightly but maintains over 90% absorption.

The metamaterial property is also vital for understanding absorption behavior. The effective refractive index (η_r_) is calculated by Equation (5):

(5) $$\eta_{r}=\pm \frac{1}{\mathrm{kd}}\cos_{}^{-1}\left[\frac{1}{{2S}_{21}}(1-S_{11}^{2}{+S}_{21}^{2})+2n\pi \right]$$

where k=2πλ/c, c is the velocity of light, S_11_ is the reflection coefficient, S_21_ is the transmission coefficient, n is the integer number and d is the substrate thickness. Equations (6) and (7) [36] are also used to retrieve the relative permittivity (ε_r_) and relative permeability (µ_r_), where Z_r_ is the relative impedance.

(6) $$\varepsilon_{r}=\frac{\eta_{r}}{Z_{r}}$$

(7) $$\mu_{r}{=n}_{r}Z_{r}$$

**Figure 14:**
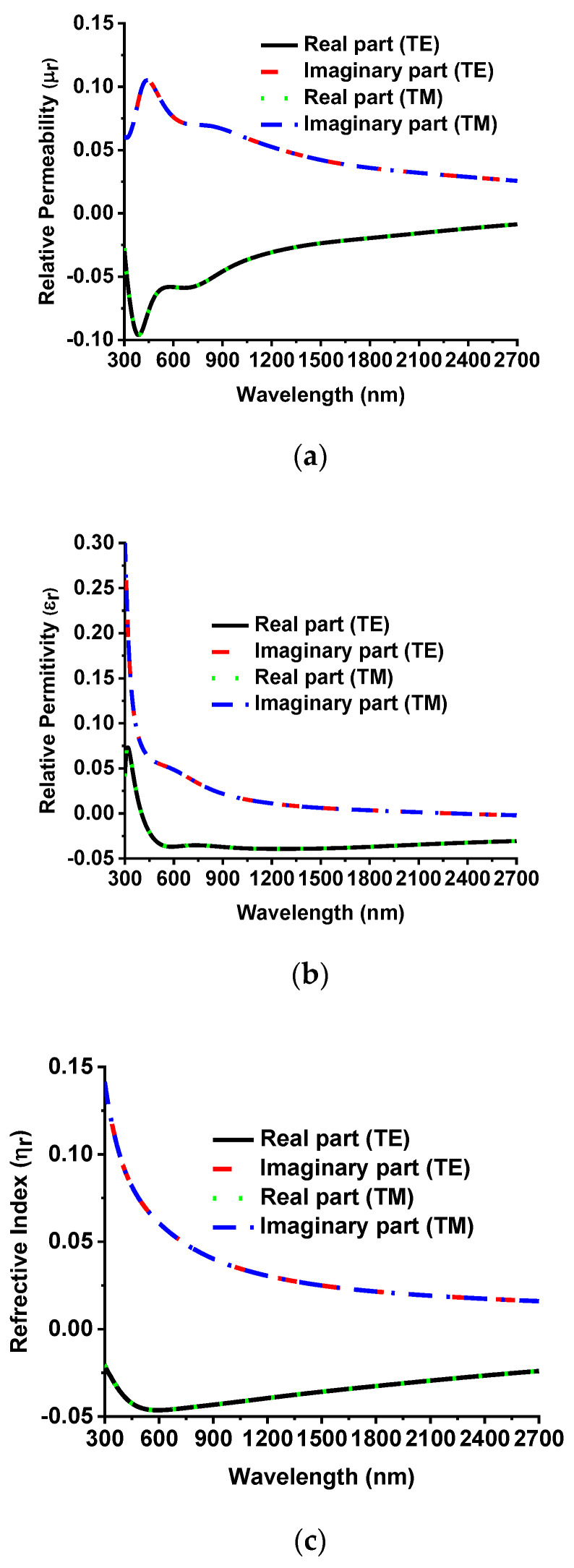
Effective EM parameter. (**a**) Relative permeability (µ_r_), (**b**) relative permittivity (ε_r_) and (**c**) for both the effective refractive index for TE and TM mode.

The imaginary and real values of the relative permeability (µ_r_), relative permittivity (ε_r_) and effective refractive index (η_r_) of the designed MMA for both TM and TE modes are illustrated in Figure 14a–c. The imaginary parts of the effective EM parameters are positive in the absorption band, indicating The dissipation impact of the incident electromagnetic wave indicated by positive imaginary part of effective EM parameters at the absorption spectrum. Furthermore, the magnetic and electric resonance occurs from the effective EM parameters because it is seen that (μ_r_), and (ε_r_) were mutated near resonance peaks, which helps to improve the absorbing strength and increases broadband absorption.

## 5. Comparison

The comparison of proposed work with existing research has been distinguished in Table 1 in terms of the material used, dimensions, operational wavelength and average absorption, peak absorption, bandwidth, MMA configuration, etc. The results show that the proposed work offers significant improvements in operational bandwidth, which covers the total visible and infrared window, where [28,37,38,39,40] covers only the visible range, and only a portion of infrared wavelength is covered by [41,42,43] and [36,44] covers a portion of infrared and visible wavelength. The average absorption level also significantly increases more than all the listed work in Table 1 for the visible and infrared spectrum. Moreover, the size of the unit cell is significantly reduced. Finally, width and high absorption bandwidth over the visible and infrared region with polarization-insensitive behavior and oblique incident-angle stability make the proposed Ni-AlN-Ni-based hexagonal ring MMA attractive in the absorption energy-harvesting application field for any polarization of light, etc.

## 6. Conclusions

This article proposed an ultrathin concentric hexagonal ring resonator (CHRR)-based metamaterial absorber for optical window applications. The overall size of the three-layered (Ni-AlN-Ni) MMA unit cell is 66 × 60 × 34 nm^3^. The designed MMA exhibits strong absorptivity in the visible and infrared region with 96.3% average absorption from 380–2700 nm wavelength. The maximum absorption of 99.98% is attained at 572 nm, with above 90% absorption realized from 380–2700 nm. The proposed metamaterial absorber offers polarization-independent properties and stable oblique incidence angles for both TM and TE polarization. The analysis of metamaterial property, magnetic field, electric field, and surface current distribution is investigated to understand the absorption mechanism. Finally, a detailed comparison with the state of the art is performed in Table 1, which shows better acceptability of the proposed MMA over others in terms of unit cell size, absorption level, operational wavelength, etc. Therefore, the proposed MMA has great potential for applications of thermal emitters, infrared sensors, energy harvesters, etc.

## Figures and Tables

**Table 1:** Comparison table.

Reference	Material	Dimension (nm^3^)	Operation Band (nm)	Average Absorption	Absorption Pick (nm)	MMA Configuration	Polarization-Insensitive	Year
[28]	W, SiO_2_	950 × 950 × 240	380–750	97.1%	99.99% at 521.83 nm	Square ring	Yes	2022
[36]	Ni, SiO_2_	200 × 200 × 75	200–2000	80%	98% at 820 nm and 2300 nm	7 hexagonal nano-rings	N/A	2021
[37]	SiO_2_, Au, Ag, Al and Ni	800 × 800 × 130	400–700	92%	98% at 470 nm	Multilayer half-cylindrical shape	Yes	2020
[38]	rs43 SiO_2_ and Al	400 × 400 × 650	435–615	-	99% at 460 nm, 520 nm and 570 nm	4 stripes and 4 square resonators	No	2020
[39]	W, SiO_2_	380 × 380 × 150	445–780	60%	99% at 798 nm	7 round nanodisks	N/A	2019
[40]	Tungsten SiO_2_	200 × 200 × 130	400–750	90%	99% at 465 nm and 530 nm	Elliptical ring resonator	No	2020
[41]	Cu- SiO_2_-Cu	220 × 220 × 300	800–1500	95%	99% at 872.54 nm and 1008.69 nm	Ring and 4 capacitor plate resonators	Yes	2020
[42]	Ti, Ge	1400 × 1400 × 620	2000–14000	87%	99% at 10490 nm	4-layer single nanoring	Yes	2020
[43]	Ti/Ge/ S$i_{3}N_{4}$	4000 × 4000 × 2300	2000–14000	78%	98.6% at 8530 nm	Periodic square resonators	Yes	2021
[44]	SiO_2_, Ti, MgF_2_, Au, TiO_2_	200 × 190 × 545	500–1700	82%	99.5% at 589 nm and 1097 nm	6-layer four-square resonator	Yes	2022
Proposed	Ni Aluminium Nitride	66 × 66 × 35	380–2500	96%	99.99% at 618 nm	Hexagonal ring resonators	Yes	2022

## Data Availability

The data presented in this study are presented in this article.
